# Individual Placement and Support supplemented with cognitive remediation and work-related social skills training in Denmark: study protocol for a randomized controlled trial

**DOI:** 10.1186/s13063-015-0792-0

**Published:** 2015-06-21

**Authors:** Thomas Nordahl Christensen, Iben Gammelgaard Nielsen, Elsebeth Stenager, Britt Reuter Morthorst, Jane Lindschou, Merete Nordentoft, Lene Falgaard Eplov

**Affiliations:** Mental Health Center Copenhagen, Mental Health Services Capital region of Denmark, University of Copenhagen, Kildegårdsvej 28, Opg.15.4, DK-2900 Hellerup, Copenhagen Denmark; Department of Psychiatry, Odense University Hospital, Sdr. Boulevard 29, DK-5000 Odense C, Denmark; Copenhagen Trial Unit, Centre for Clinical Intervention Research, Rigshospitalet, Department 7812 Copenhagen University Hospital, Blegdamsvej 9, DK-2100 Copenhagen Ø, Denmark

**Keywords:** Individual placement and support, Supported employment, Cognitive remediation, Social skills training, Severe mental illness, Competitive employment, Competitive education

## Abstract

**Background:**

Individual Placement and Support (IPS) appears to be an effective vocational intervention for obtaining competitive employment for people with severe mental illness. However, no IPS studies or trials have been conducted in Denmark, a country characterized by a specialized labor market with a higher minimum wage and fewer entry-level jobs in comparison with other countries such as the US. Furthermore, long-term job retention and economic self-sufficiency have not been clearly demonstrated. Integrating methods such as cognitive remediation and work-related social skills training may be ways to address these issues.

**Methods/Design:**

The trial design is an investigator-initiated, randomized, assessor-blinded, multi-center trial. A total of 750 patients with severe mental illness will be randomly assigned into three groups: (1) IPS, (2) IPS enhanced with cognitive remediation and work-related social skills training, and (3) service as usual. The primary outcome is number of hours in competitive employment or education at 18-month follow-up. Secondary and exploratory outcomes are money earned, days to first employment, symptoms, functional level, self-esteem, and self-efficacy at 18-month follow-up. Thirty- and 60-month follow-ups will be register-based.

**Discussion:**

This will be one of the largest randomized trials investigating IPS to date. The trial will be conducted with high methodological quality in order to reduce the risk of bias. If the results of this trial show that IPS, or IPS enhanced with cognitive remediation and work-related social skills training, is superior to service as usual, this will support preliminary evidence. Furthermore, it will show that the method is generalizable to a variety of labor markets and welfare systems and provide important knowledge about the effect of adding cognitive remediation and social skills training to the IPS intervention.

**Trial registration:**

ClinicalTrials registration number: NCT01722344 (registered 2 Nov. 2012).

## Background

People with severe mental illnesses, defined as psychotic disorders, bipolar disorders, or major depression, identify employment or education as a key component to their recovery process, and approximately 65 % endorse employment as a goal [1–3]. However, employment seems to be a challenge in this population in which previous research has estimated a global unemployment rate of up to 90 %, which results in both personal and socioeconomic costs [4, 5].

Conventional vocational rehabilitation programs meet these challenges by employing a “train and place” approach, emphasizing prevocational training such as sheltered employment or trainee placements [1]. This approach remains the most widespread but has been shown to have very poor effects on competitive employment as well as low rates of client retention [6, 7].

In contrast, Individual Placement and Support (IPS) follows a “place and train” philosophy, which consists of an individualized and rapid search for competitive employment or education, avoiding prolonged prevocational training and preparation [8–10]. The intervention is integrated within the mental health services with emphasis on client preferences and choice regarding jobs and includes ongoing job support and benefit counseling [8–10].

The effects of the IPS intervention have been investigated in a number of randomized clinical trials, and reviews of these trials suggest that IPS is superior to other types of vocational rehabilitation programs in regard to obtaining competitive employment [6–9]. A review including 15 high-fidelity IPS trials shows an average employment rate among the IPS participants of 58.9 % compared with 23.2 % for control participants [7]. All of the control groups consisted of either treatment as usual, typically referral to the state vocational system, or well-established alternative vocational models [7]. Outcomes related to wages earned and hours worked were also found to be superior among those receiving the IPS intervention [7]. Previous research has not found that IPS leads to increased stress, exacerbation of symptoms, or other harmful clinical outcomes [8, 10]. The results of a recent Cochrane systematic review investigating IPS-supported employment for adults with severe mental illness additionally suggest that, compared with other vocational approaches, IPS is effective in improving a number of vocational outcomes relevant to people with severe mental illness [11]. However, the authors conclude that evidence from the included randomized trials was of “very low quality” mainly due to a high risk of bias (i.e., not describing allocation concealment). Furthermore, the meta-analysis excluded a majority of previous trials because of skewed data and populations of fewer than 200 people [11].

Moreover, it has been suggested that long-term job retention and economic self-sufficiency could be further improved by adding cognitive remediation and work-related social skills training to the intervention [10, 11]. Impairment in these functions is frequent among persons receiving IPS services and is known to be related to employment outcomes in persons with severe mental illness [12–14]. Two small-scale trials have found improved effects when the intervention was enhanced with either cognitive remediation or work-related social skills training [13, 15–17].

Randomized clinical trials of IPS have been conducted in different socioeconomic and cultural contexts with different results. A review found that four of five trials with the lowest employment rates were non-US trials [7]. Furthermore, a randomized trial from the UK investigating IPS compared with service as usual did not show significant vocational effects [18]. The authors suggest that implementation of IPS in labor markets and economies where economic disincentives may lead to lower levels of motivation can be challenging [18]. Until now, IPS trials have not been conducted in Denmark, where barriers to implementation and replication of previous international findings may exist. Firstly, Denmark is characterized by complex employment legislation and a highly specialized labor market with a high minimum wage and few entry-level jobs, which could impact both the implementation of IPS and potential effect sizes. Secondly, the social security system is generous compared with those of other countries, and this may be a perceived or real financial disincentive for returning to competitive employment and hence influence motivation levels [18, 19].

Thus, it is crucial to investigate whether IPS can be implemented in Denmark, and a large-scale trial with low risk of bias and a long follow-up period is needed. The present trial will be the largest randomized clinical trial to date to investigate the effects of IPS and IPS enhanced with cognitive remediation and work-related social skills training. The primary outcome is number of hours in competitive employment or education at 18-month follow-up.

## Methods/Design

### Trial design

The trial is designed as an investigator-initiated, randomized, three-arm, assessor-blinded, multi-center trial. A total of 750 patients with severe mental illness will be randomly assigned into (1) IPS, (2) IPS enhanced with cognitive remediation and work-related social skills training, and (3) service as usual.

The primary hypothesis is that participants allocated to the IPS intervention group (group 1) will have significantly higher work or study rates at 18-month follow-up compared with participants allocated to service as usual (group 3). Furthermore, we assume that an enhancement of the IPS intervention with cognitive remediation and work-related social skills training (group 2) will increase the effects. To ensure high methodological quality, the trial is designed and reported according to the SPIRIT (Standard Protocol Items: Recommendations for Interventional Trials) Statement and the modified CONSORT (Consolidated Standards Of Reporting Trials) criteria for non-pharmacological trials [20, 21].

### Recruitment and eligibility criteria

Eligible participants are adults (ages 18–67) diagnosed according to the International Classification of Diseases version 10 (ICD-10) with schizophrenia, schizotypal, or delusional disorders (F20–F29); or bipolar disorder (F31); or severe depression (F33). Participants must reside in one of two major Danish cities: Copenhagen (including the municipality of Frederiksberg) or Odense. They must be assigned to early intervention teams (OPUS teams) or community mental health services at Mental Health Center Copenhagen or the Department of Mental Health Odense-University Clinic. They must express a clear desire for competitive employment or education and provide verbal and written informed consent. Furthermore, participants must be able to speak and understand Danish well enough to participate without an interpreter, mainly in order to benefit from the group-based cognitive remediation therapy. A connection to vocational authorities with a formalized collaboration with the IPS teams is the foundation for the residence criterion. If this criterion leads to an insufficient number of patients, expansion of the geographic area of the trial will be considered. Participants who are interested in competitive employment or education are identified by case managers, who assess for eligibility and refer to the trial. To ensure that the participants meet the diagnostic criteria, they will be assessed by a trained and certified research assistant using the Schedules for Clinical Assessment in Neuropsychiatry (SCAN) diagnostic tool [22]. Informed consent will be obtained from each participant before assessment.

### Randomization

After the assessment, a central web-based randomization will be performed by the Copenhagen Trial Unit [23] according to a computer-generated allocation sequence with permuted blocks of varying sizes. The allocation sequence and varying block sizes will be concealed from the investigators. A research secretary will perform the allocation by logging on to a website by using a personal password. Previous research has shown an effect of sex and of work history on vocational outcomes [24, 25]. Therefore, the allocation sequence will be stratified by sex and by work history (more or less than 2 months of competitive employment during the last 5 years). Furthermore, participants will be stratified by work readiness by using match categories, a tool used in Danish job centers [26]. We will assess the likelihood that the participant is ready to apply for competitive employment and will be self-sufficient within 3 months (match 2 or 3). Finally, the participants will be stratified by site.

### The experimental interventions

#### Group 1 – IPS

The first experimental intervention group will receive IPS and service as usual (see Control group below). The details of the IPS intervention are described comprehensively in the IPS literature [1, 27] and are briefly outlined below with emphasis on the specific challenges and opportunities for implementation into a Danish context. The IPS intervention is based on eight key principles: (1) eligibility based on client choice, (2) focus on competitive employment or education, (3) integration of mental health and employment services, (4) attention to client preferences, (5) benefits counseling, (6) rapid job search, (7) systematic job development, and (8) individualized long-term job support [1, 7]. Competitive employment is defined as part-time or full-time jobs that exist in the open labor market and that pay at least a minimum wage and are open to everyone, regardless of their disability status [9]. Competitive education is defined as an education or training program that is related to an employment goal and not designed specifically for people with disabilities [27].

Danish employment legislation provides opportunities for financial support when obtaining competitive employment. This could be subsidized employment that in most cases will be consistent with the definition of competitive employment, as it consists of jobs that pay at least the minimum wage and are open to everyone. For more information, see Table 1.

**Table 1:** Danish employment legislation

In Denmark, subsided employment at a private workplace is offered to long-term unemployed individuals with or without a disability. Working conditions are agreed upon between authorities and employers and are formalized in a contract. The employee in a private workplace will receive at least minimum wage. Maximum duration of a subsidized job is 1 year, although many will continue in regular employment without subsidy if agreed upon with the employer. Participants will also receive help finding a fleksjob if this is already granted at intake to the study. Fleksjob is also subsidized employment, but the subsidy is conditional on the employee’s ability to work. The employee will receive at least minimum wage for the actual hours of work. The job exists on the open labor market.
Employment specialists in Copenhagen are employed by the vocational authorities in the municipality (job centres) but will still be integrated within the mental health services.

It is expected that many of the participants in the trial will have an aim or motivation to start or resume education. This is expected, firstly, because many of the participants will be young adults recruited from early intervention teams (OPUS) and, secondly, because the education system in Denmark is financed by the state or the municipalities without tuition fees and with the opportunity to receive financial support from the State Educational Grant and Loan Scheme (SU). These options give employment specialists greater opportunities to focus on education compared with previous IPS reports. Employment specialists are encouraged to closely follow the methods described in the updated and expanded IPS manual “Applying the individual placement and support (IPS) model to help clients compete in the workforce” [27]. The manual, including worksheets, is translated into Danish, and the two IPS teams will be trained in the method by an IPS expert, who will also offer tele-supervision throughout the trial period. The IPS employment specialists will be evaluated by trained external reviewers who will use the IPS fidelity scale to ensure high fidelity and adherence to evidence-based practice [28]. The evaluation will take place 6 months after trial start and thereafter every sixth month until high fidelity is demonstrated. Subsequently, an annual evaluation will be performed.

#### Group 2 – IPS enhanced with cognitive remediation and work-related social skills training

The second experimental intervention group will receive IPS (see Group 1 – IPS) enhanced with cognitive remediation and work-related social skills training and service as usual (see Control group below). The cognitive remediation is, with a few adjustments, based on previous research by McGurk et al. [15] and uses an adapted version of the “Thinking skills for work” manual [13, 15]. This is designed as an adjunct to IPS and is aimed at integrating cognitive rehabilitation with the ongoing provision of IPS services. The enhancement program consists of 24 group-based sessions of computer training using newly developed software (CIRCuiTS) and incorporates evidence-based training principles such as errorless learning and massed practice [29]. The computer training provide practices across a broad range of cognitive functions hypothesized to be impaired in persons with severe mental illness, including attention, concentration, psychomotor speed, learning, memory, and executive functions. Each participant works through a so-called metacognitive journey consisting of 278 task instances divided into seven different stages. The participant receives ongoing feedback and is able to monitor their own scores, strategy use, progression in skills, and development or change in personal goals. In addition to receiving the computer training, participants are offered 12 sessions in coping strategies for dealing with cognitive challenges [13]. These sessions are aimed at helping participants develop effective strategies for improving their cognitive skills or reducing the effects of cognitive challenges in order to achieve vocational goals, maintain work, and increase performance [13]. Finally, the program consists of six work-related social skills training sessions with a focus on disclosure, communications skills, decoding norms for social interaction, and conflict management. A detailed manual based mainly on the “Thinking skills for work” manual was developed in Danish but was adapted to the present trial and extended with work-related social skills training. The manual has not been published but can be obtained by request from the authors. The Danish manual further deviates by providing less opportunity for individual training and by implementing CIRCuiTS instead of Cogpack computer software.

The intervention will be performed primarily in group format, and eight participants will be assigned to each group. Trained psychologists will be responsible for the group sessions, and employment specialists will be co-therapists. The program requires 30 weeks to complete, is complementary to IPS, and should not be considered prevocational training. While participating in groups, participants will seek regular employment or education. To ensure the quality of the intervention, employment specialists are trained by psychologists with experience in using the method. A fidelity scale of the intervention has been developed, and fidelity will be assessed at the same time as the IPS fidelity review.

### Control group

#### Group 3 – service as usual

Participants allocated to the control group will receive “service as usual” only. This consists of participants continuing to receive OPUS or community mental health treatment. Also, it involves individual case management and medical review, referral to external vocational agencies, and involvement in group programs which may involve participation in vocationally oriented groups. The psychiatric treatment provided will be the same in all three groups throughout the trial period with the one exception that controls do not get the integrated IPS intervention. In general, the participants allocated to the control group are to have close mandatory contact with the local vocational authority (job centers), depending on what kind of benefits they receive. Hence, the participants in the control group will receive a variety of vocational rehabilitation support at the job centers in accordance with the train and place principle.

### Blinding

Owing to the nature of the intervention, neither participants nor staff can be blinded to allocation but are instructed not to reveal details that may cause the research assistant to deduce which intervention the participants are receiving. The research assistants who perform the assessments at baseline and follow-up will be blind to allocation. If the blinding cannot be maintained, a research assistant from the other site will perform the follow-up interviews. Blinding will be maintained until the end of the trial. Statistical analyses will be conducted with intervention groups coded as, for example, X, Y, and Z. Conclusions will be drawn with the blinding intact. First, we will assume that X is experimental group 1, Y is experimental group 2, and Z is control group 3. Then we will draw five additional conclusions, assuming the remaining five combinations. After this, the blind will be broken.

### Outcome and assessments

The primary outcome is “hours in competitive employment or education” measured from baseline to 18-month follow-up. Employment and enrollment in education will be identified by using register data. Hours in competitive employment will be extracted from an extended version of the Danish Register for Evaluation of Marginalization (DREAM) database administered by the National Labor Market Authority [30]. The DREAM database contains information on employment, sickness leave, and education eligible for state education grants, disability pension, social security, and sickness benefits. The register covers the entire population, and data can be linked to a range of different registers, including the Danish income register, making it possible to obtain the exact number of employment hours. Data on education will be extracted from education statistics hosted by Statistics Denmark (http://www.statbank.dk) and supplemented by data from interviews for more detailed information on part-time studies.

The primary outcome will be supported by several other secondary and explorative outcome measures. Secondary outcomes are work or education at some point during the follow-up period (yes/no), days to first employment or beginning of education, cognitive impairment, functional level, self-esteem, and self-efficacy assessed at baseline and 18-month follow-up. Data on employment and education will similarly be extracted from registers. Furthermore, semi-structured interviews using the Personal and Social Performance (PSP) scale [31] and the Brief Assessment of Cognition in Schizophrenia [32] will be used to assess cognitive impairment and functioning. Standardized validated survey instruments, including the Rosenberg self-esteem scale [33] and the General Self-Efficacy scale [34], will be used to assess self-esteem and self-efficacy. All secondary outcomes and the assessment instruments used are outlined in Table 2.

**Table 2:** Primary and secondary outcomes and data collection

Outcomes	Source of collection	Assessment	Baseline	18-month follow-up
Primary outcome	Register-based/Interview	Hours in competitive employment or education in follow-up period		X
Secondary outcomes	Register-based	Competitive employment or education at some point during follow-up period		X
	Register-based	Days to first employment or beginning of education		X
	Obtained through interview	Cognitive function measured with the Brief Assessment of Cognition in Schizophrenia [32] (BACS)	X	X
	Obtained through interview	Function measured with Personal and Social Performance (PSP) Scale [31]	X	X
	Self-reported questionnaire	Self-efficacy measured with General Self-Efficacy (GSE) scale [37]	X	X
	Self-reported questionnaire	Self-esteem measured with the Rosenberg Self-Esteem scale [38]	X	X

To avoid the risk of multiplicity and type I errors, we will limit our secondary outcomes to outcomes with at least 80 % power. Other outcomes are considered “exploratory” when drawing conclusions. This means that we will consider any statistically significant result in any of these outcomes as exploratory or hypothesis-generating. This is because we have no sample size estimation for these outcomes, and thus the risk of an early false positive is increased; it is further increased because of the risk of multiplicity as a result of too many outcomes.

Exploratory outcomes cover the 30 and 60 months of register follow-up along with additional vocational measures that will provide a more nuanced picture of vocational status, including average monthly earnings and hours of work per week among those who obtain competitive employment. Furthermore, a range of non-vocational outcomes such as client satisfaction, health-related quality of life, empowerment, recovery, and substance abuse will be used to address other important factors hypothesized to influence the participants. Psychopathology will be measured to ensure no adverse effects of the intervention.

All exploratory and safety measures and the assessment instruments used are outlined in Tables 3 and 4. Trained and certified research assistants will perform all assessments. Inter-rater reliability tests will be performed prior to the trial and at least quarterly throughout the assessment period on the following instruments: Scale for the Assessment of Negative Symptoms [35], Scale for the Assessment of Positive Symptoms [35], PSP scale [31], and Hamilton Depression Scale (HAM-D6) [36]. The aim is to achieve an interclass correlation coefficient of more than 0.7. Consensus ratings will be performed on the remaining instruments.

**Table 3:** Explorative measures and data collection

Outcomes	Source of collection	Assessment	Baseline	18-month follow-up	30 + 60-month register follow-up
Explorative outcomes	Register-based	Hours in competitive employment or education in follow-up period			X
	Register-based	Competitive employment or education at some point during follow-up period			X
	Register-based	Days to first employment or beginning of education			X
	Register-based	Days in employment or education		X	X
	Register-based	Average monthly earnings		X	X
	Register-based	Hours of work per week among those who obtain competitive employment		X	X
	Register-based	Long-term sick leave		X	X
	Register-based	Social benefits	X	X	X
	Obtained through interview	Function measured with Global Assessment of Functioning (GAF) [39]	X	X	
	Obtained through interview	Health-related quality of life measured with 12-Item Short Form Health Survey (SF-12) [40]	X	X	
	Self-reported questionnaire	Recovery measured with Mental Health and Recovery Measure (MHRM) [41]		X	
	Self-reported questionnaire	Empowerment measured with Empowerment Scale [42]	X	X	
	Self-reported questionnaires	Satisfaction with treatment. Measured with Client Satisfaction Questionnaire (CSQ) [43]		X	
	Register-based	Use of mental health services	X	X	X
	Obtained through interview	Substance abuse measured with Alcohol Use Disorders Identification Test (AUDIT) [44]	X	X	
	Self-reported questionnaire	Health-related quality-of-life measured with EQ 5D(EuroQOL five dimensions questionnaire) [45]	X	X

**Table 4:** Safety measures and data collection

Outcome measure	Source of collection	Assessment	Baseline	18-month follow-up	30 + 60-month register follow-up
Safety measures	Obtained through interview	Psychotic and negative symptoms measured with Scale for the Assessment of Positive Symptoms (SAPS) and Scale for the Assessment of Negative Symptoms (SANS)	X	X	
	Obtained through interview	Depressive symptoms measured with Hamilton Depression Scale [36] (HAM-D6)	X	X	
	Obtained through interview	Suicidal ideation and actions. Measured with European Parasuicide Study Interview Schedule (EPSIS)	X	X	
	Death cause register, Civil Registration System (CPR)	Deaths (all causes)	X	X	X
	Hospital records	Number and length of hospital admissions both somatic and psychiatric		X	X
	Hospital records	Use of outpatient services	X	X	X

### Adherence to the interventional program

The employment specialists and the IPS team leaders will assess the use of IPS services, including content and number of contacts between employment specialist and participants. The cognitive specialists will register adherence to the cognitive remediation and social skill groups, including group session attendance and whether home work is completed. In the control group, all contact between the participants and the social worker or vocational counselor will be assessed. It will also be possible to assess whether the controls are allocated to a vocational rehabilitation program of any kind and the duration and content of the rehabilitation. These data will be obtained from the DREAM register and from records in the job centers.

### Sample size and power calculation

No previous IPS trials or similar studies have been conducted in Denmark that could contribute to an estimation of the expected average number of hours of work among participants in the IPS intervention. A European IPS multicenter trial found a difference between the IPS group and the control group (vocational services) of 150 h in competitive employment in an 18-month follow-up period, and standard deviation was 500 [19]. A difference of 150 h in competitive employment or education is considered clinically relevant. If the outcome within each intervention group is normally distributed with a standard deviation on 500 and with a true mean difference of 150 between the intervention and the control group, the present trial will need 250 participants in both intervention arms and additionally 250 control participants to be able to reject the null hypothesis that the population means of the experimental and control groups are equal with probability (power) of 80 %. The type I error probability associated with the null hypothesis is 1.25 %. A type I error of 1.25 % was chosen to give the possibility to make four comparisons: (1) IPS versus service as usual, (2) IPS versus IPS enhanced, (3) IPS enhanced versus service as usual, and (4) IPS + IPS enhanced versus service as usual. If we encounter difficulties in recruiting 250 participants to each group, we will exclude comparison (4) as a primary comparison. With three primary comparisons, we use a type I error of 1.67 % in the sample size calculation, accumulating a sample size of 236 patients in each group. Power calculations on calculations of the secondary outcome measures were carried out (Table 5) and indicate that a sample size of 250 patients per group would be adequate to detect relevant significant differences.

**Table 5:** Power calculation

Outcome measure	δ expected difference in mean	σ expected standard deviation	α	Power	Reference
Days to first employment or beginning of education	68	45	0.0125	1.000 (*t* test)	Bond et al. [8] (2008),
Cognitive function	0.3	0.7	0.0125	0.989 (*t* test)	McGurk et al. [13] (2005)
The Brief Assessment of Cognition in Schizophrenia [32]					
Function measured with Personal and Social Performance (PSP) scale [31]	7	14	0.0125	0.999 (*t* test)	Kawata et al. [31] (2008). Nasrallah et al. [46] (2008) (No IPS studies use PSP scale. δ is estimated)
Self-efficacy measured with General Self-Efficacy scale [37]	0.28	0.85	0.0125	0.880 (*t* test)	Tsang et al. [14] (2010)
Self-esteem measured with the Rosenberg Self-Esteem scale [38]	0.3	0.55	0.0125	0.992 (*t* test)	Mueser et al. [47] (2004), Howard et al. [18] (2010), Drake et al. [48] (1999). None of the studies shows difference in mean. σ is between 0.55 and 0.68.
Competitive employment or education during follow-up period	50 % vs. 34 %	-	0.0125	0.807 (chi-squared test)	Bond et al. [8] (2008)
Dichotomous yes/no					

### Data analysis plan

The main null hypothesis to be tested is that there is no difference between the three groups in hours in competitive employment or education in the 18-month follow-up period. All randomized participants will be analyzed, including those who stop receiving treatment, according to the intention-to-treat principle. All continuous outcome measures, including the primary outcome “hours in competitive employment or education”, will be analyzed by using generalized linear models. In situations in which the continuous measure is non-normally distributed, a non-parametric model will be used. Multiple imputations will be used to impute a distribution of missing values. Furthermore, linear mixed models with repeated measurements and unstructured covariance matrixes will be used to assess the potential interaction between time and intervention. The dichotomous secondary outcome “work or education at some point during the follow-up period” will be analyzed by using logistic regression. The secondary outcome “time-to-employment or education” will be analyzed by using the Cox proportional hazards model. All models will be adjusted for the stratification variables.

### Ethical considerations

Previous research does not indicate that IPS leads to an exacerbation of symptoms or has other negative clinical implications [10]. Entering competitive employment may, for some participants, be perceived as stressful. This will be addressed by close contact with participants, case managers, and employers to ensure adaptation of special requirements or sick leave if necessary. All adverse events (e.g., hospitalization, increase in symptoms, decrease in functioning, and incidents of suicide) will be registered and reported. All safety measures can be seen in Table 4.

Information about the trial is presented to all potential participants both verbally and in written form so they can make an informed decision about their participation before signing written consent. It will be clearly explained that participation is voluntary and that withdrawal can occur at any time without consequence for treatment possibilities. Decisions regarding participation will not influence clinical care in any way.

The trial protocol has been reviewed by the Ethics Committee in the Capital Region of Denmark (registration #H-3-2012FSP34), although they waived the need for ethical approval because it is not a biomedical trial. Furthermore, the trial has been reported to the Danish Data Protection Agency (registration #01768 RHP-2012-011) and is registered at http://www.clinicaltrials.gov (#NCT01722344).

## Discussion

This is the first trial investigating the effects of IPS in Denmark and will be one of the largest randomized trials investigating IPS to date. As a comparison, 14 trials included in a Cochrane review showed a median sample size per arm of 70 participants and a range of between 20 and 156 [11]. Furthermore, this trial will be the first large-scale trial, with a long follow-up period, enhancing the IPS intervention with both cognitive remediation and social skills training. The design of the trial has several strengths. Firstly, a sample size calculation was made according to the primary outcome, hours in competitive employment or education. The power is estimated for all secondary outcome measures, showing that a sample size of 750 participants is sufficient to show a relevant effect size with a power above 80 %. Secondly, the risk of selection bias related to allocation sequence generation and concealment is limited, as the Copenhagen Trial Unit performs a central web-based randomization according to a computer-generated allocation sequence. Thirdly, assessors are blinded, and blinding will be used wherever possible to prevent bias. Data will be analyzed according to the intention-to-treat principle, which together with an intense follow-up of patients should limit the risk of attrition bias. Finally, internal validity is addressed by implementing fidelity ratings. The fidelity ratings have high priority and will be conducted throughout the trial period to ensure that evidence-based practice is adhered to.

The trial also has some limitations. Firstly, there is a risk of performance bias because participants and practitioners are not blinded. It could be argued that both participants and practitioners conducting the experimental intervention would be more enthusiastic and keen to perform well because of the novelty of participating in a research project. To account for this, blinding is employed in all other aspects of the trial. Secondly, the control condition is heterogeneous. Participants will receive different vocational interventions, which will vary by individual and depend on factors such as type of social benefit the individual is receiving. Policy decisions could change the conditions in the job centers during the trial period and this could affect vocational rehabilitation, a fact that may limit the generalizability of the control condition. Thirdly, recruitment procedures may affect the external validity of the trial. The staff at the mental health centers is responsible for recruitment and may not successfully identify all eligible participants. Finally, there are initiatives in the Danish IPS model that exclude the possibility of highest IPS fidelity score. For example, the employment specialists will use a percentage of their time on the authority work in the job centers instead of manualized IPS.

If the results of this trial show IPS to be effective compared with the control group, these positive results will support the preliminary evidence that the method is generalizable to a variety of sociodemographic contexts. Furthermore, if IPS supplemented with cognitive remediation and work-related social skills training shows that the effects can be further improved, it will bring important knowledge for further research on and implementation of IPS.

## Trial status

The trial was initiated in October 2012. As of January 2015, 480 patients had been randomly assigned.

## Abbreviations

CIRCuiTS: Computerised Interactive Remediation of Cognition - Training for Schizophrenia
DREAM: Danish Register for Evaluation of Marginalization
IPS: Individual Placement and Support
PSP: Personal and Social Performance (scale)

## References

[1] Drake RE, Bond GR, Becker DR (2012). Individual placement and support: an evidence-based approach to supported employment.

[2] Bond GR, Drake RE (2014). Making the case for IPS supported employment. Adm Policy Ment Health.

[3] Bond G (2004). Suported employment: evidence for an evidence-based practice. Psychiatr Rehabil J.

[4] Marwaha S, Johnson S, Bebbington P, Stafford M, Angermeyer MC, Brugha T (2007). Rates and correlates of employment in people with schizophrenia in the UK, France and Germany. Br J Psychiatry.

[5] Harnois G, Gabriel P (2000). Mental health and work: impact, issues and good practices.

[6] Crowther RE, Marshall M, Bond GR, Huxley P (2001). Helping people with severe mental illness to obtain work: systematic review. BMJ.

[7] Bond GR, Drake RE, Becker DR (2012). Generalizability of the Individual Placement and Support (IPS) model of supported employment outside the US. World Psychiatry.

[8] Bond GR, Drake RE, Becker DR (2008). An update on randomized controlled trials of evidence-based supported employment. Psychiatr Rehabil J.

[9] Campbell K, Bond GR, Drake RE (2011). Who benefits from supported employment: a meta-analytic study. Schizophr Bull.

[10] Dixon LB, Dickerson F, Bellack AS, Bennett M, Dickinson D, Goldberg RW (2010). The 2009 schizophrenia PORT psychosocial treatment recommendations and summary statements. Schizophr Bull.

[11] Kinoshita Y, Furukawa TA, Kinoshita K, Honyashiki M, Omori IM, Marshall M (2013). Supported employment for adults with severe mental illness. Cochrane Database Syst Rev.

[12] McGurk SR, Meltzer HY (2000). The role of cognition in vocational functioning in schizophrenia. Schizophr Res.

[13] McGurk SR, Mueser KT, Pascaris A (2005). Cognitive training and supported employment for persons with severe mental illness: one-year results from a randomized controlled trial. Schizophr Bull.

[14] Tsang HW, Leung AY, Chung RC, Bell M, Cheung WM (2010). Review on vocational predictors: a systematic review of predictors of vocational outcomes among individuals with schizophrenia: an update since 1998. Aust N Z J Psychiatry.

[15] McGurk SR, Mueser KT, Feldman K, Wolfe R, Pascaris A (2007). Cognitive training for supported employment: 2–3 year outcomes of a randomized controlled trial. Am J Psychiatry.

[16] Tsang HW, Chan A, Wong A, Liberman RP (2009). Vocational outcomes of an integrated supported employment program for individuals with persistent and severe mental illness. J Behav Ther Exp Psychiatry.

[17] Tsang HW, Fung KM, Leung AY, Li SM, Cheung WM (2010). Three year follow-up study of an integrated supported employment for individuals with severe mental illness. Aust N Z J Psychiatry.

[18] Howard LM, Heslin M, Leese M, McCrone P, Rice C, Jarrett M (2010). Supported employment: randomised controlled trial. Br J Psychiatry.

[19] Burns T, Catty J, Becker T, Drake RE, Fioritti A, Knapp M (2007). The effectiveness of supported employment for people with severe mental illness: a randomised controlled trial. Lancet.

[20] Boutron I, Moher D, Altman D (2008). Extending the CONSORT statement to randomized trials of nonpharmacologic treatment: explanation and elaboration. Ann Intern Med.

[21] Chan A, Tetzlaff JM, Gøtzsche PC, Altman DG, Mann H, Berlin JA (2013). SPIRIT 2013 explanation and elaboration: guidance for protocols of clinical trials. BMJ.

[22] Wing JK, Babor T, Brugha T, Burke J, Cooper JE, Giel R (1990). SCAN: Schedules for Clinical Assessment in Neuropsychiatry. Arch Gen Psychiatry.

[23] Copenhagen Trial Unit Center for clinical intervention research, 2015. http://www.ctu.dk/.

[24] Wewiorski NJ, Fabian ES (2004). Association between demographic and diagnostic factors and employment outcomes for people with psychiatric disabilities: a synthesis of recent research. Ment Health Serv Res.

[25] Drake RE, McHugo GJ, Becker DR, Anthony W, Clark RE (1996). The New Hampshire study of supported employment for people with severe mental illness. J Consult Clin Psychol.

[26] The National Labour Market Authority in Denmark (2015). The match model [Match model, Arbejdsmarkedsstyrelsen].

[27] Swanson SJ, Becker DR (2011). Supported employment applying the Individual Placement and Support (IPS) model to help clients compete in the workforce.

[28] Bond GR, Peterson AE, Becker DR, Drake RE (2012). Validation of the Revised Individual Placement and Support Fidelity Scale (IPS-25). Psychiatr Serv.

[29] Wykes T (2008). A pilot study of a new computerised cognitive remediation programme. Vasa.

[30] The National Labour Market Authority in Denmark (2015). The DREAM database, Statistics Denmark [DREAM databasen, Danmarks Statistik].

[31] Kawata AK, Revicki DA (2008). Psychometric properties of the Personal and Social Performance scale (PSP) among individuals with schizophrenia living in the community. Qual Life Res.

[32] Keefe RS, Poe M, Walker TM, Harvey PD (2006). The relationship of the Brief Assessment of Cognition in Schizophrenia (BACS) to functional capacity and real-world functional outcome. J Clin Exp Neuropsychol.

[33] Gray-Little B, Williams VS, Hancock TD (1997). An item response theory analysis of the Rosenberg Self-Esteem Scale. Pers Soc Psychol Bull.

[34] Scholz U, Gutiérrez Doña B, Sud S, Schwarzer R (2002). Is General Self-Efficacy a universal construct?. Eur J Psychol Assess.

[35] Andreasen NC, Grove WM (1986). Evaluation of positive and negative symptoms in schizophrenia. Psychiatrie & Psychobiologie.

[36] Lecrubier Y, Bech P (2007). The Ham D(6) is more homogenous and as sensitive as the Ham D(17). Eur Psychiatry.

[37] Luszczynska A, Scholz U (2005). The general self-efficacy scale: multicultural validation studies. J Psychol.

[38] Stryker S, Rosenberg M. Conceiving the self. Contemp Sociol. 1980;383.

[39] Pedersen G, Hagtvet KA, Karterud S (2007). Generalizability studies of the Global Assessment of Functioning-Split version. Compr Psychiatry.

[40] Ware J, Kosinski M, Keller S (1996). A 12-Item Short-Form Health Survey: construction of scales and preliminary tests of reliability and validity. Med Care.

[41] Bullock W (2009). The Mental Health Recovery Measure (MHRM): updated normative data and psychometric properties.

[42] Rogers ES, Chamberlin J, Ellison ML, Crean T (1997). A consumer-constructed scale to measure empowerment among users of mental health services. Psychiatr Serv.

[43] Attkisson C (1982). The client satisfaction questionnaire: psychometric properties and correlations with service utilization and psychotherapy outcome. Eval Program Plann.

[44] Babor TF, Higgins-Biddle JC, Saunders JB, Monteiro MG (2001). The alcohol use disorders identification test: guidelines for use in primary care.

[45] Rabin R, De Charro F (2001). EQ-5D: a measure of health status from the EuroQol Group. Ann Med.

[46] Nasrallah H, Morosini P, Gagnon DD (2008). Reliability, validity and ability to detect change of the Personal and Social Performance scale in patients with stable schizophrenia. Psychiatry Res.

[47] Mueser KT, Clark RE, Haines M, Drake RE, McHugo GJ, Bond GR (2004). The Hartford study of supported employment for persons with severe mental illness. J Consult Clin Psychol.

[48] Drake RE, McHugo GJ, Bebout RR, Becker DR, Harris M, Bond GR (1999). A randomized clinical trial of supported employment for inner-city patients with severe mental disorders. Arch Gen Psychiatry.

